# Potential biomarkers for predicting outcomes in CABG cardiothoracic surgeries

**DOI:** 10.1186/1749-8090-8-176

**Published:** 2013-07-18

**Authors:** Isabel Preeshagul, Rajendra Gharbaran, Kyung Hwa Jeong, Ahmed Abdel-Razek, Leonard Y Lee, Elie Elman, K Stephen Suh

**Affiliations:** 1 The John Theurer Cancer Center, Hackensack University Medical Center, Hackensack NJ 07601, NJ, USA; 2 Cardiothoracic Surgery Division, Hackensack University Medical Center, Hackensack NJ 07601NJ, USA; 3 Division of Cardiothoracic Surgery, UMDNJ-Robert Wood Johnson Medical School, New Brunswick 08901NJ, USA

**Keywords:** Biomarker, Cardiothoracic surgery, Clinical outcome, Serum, Predictive markers

## Abstract

The variations in recovery time, complications, and survival among cardiac patients who have undergone coronary artery bypass graft (CABG) procedures are vast. Many formulas and theories are used to predict clinical outcome and recovery time, and current prognostic predictions are based on medical and family history, lifestyle, co-morbidities, and performance status. The identification of biomarkers that provide concrete evidence supporting clinical outcome has greatly affected the field of medicine, helping clinicians in many medicine sub-specialties to forecast clinical course. Recent studies have discovered biomarkers that may be used as predictors of cardiac patients’ status post-cardiothoracic surgery, and the applications are numerous. In this review, we assess currently available cardiac biomarkers as predictors of clinical outcome for post-operative CABG patients. Data were collected from various studies in which cardiac biomarkers were measured in pre-operative and post-operative CABG patients.

## Review

## Introduction

Humans exist in an environment full of stress-inducing factors, such as microorganisms, poor dietary choices, family history, or emotional hardship, which can all impact human health. Our successful survival has depended on effective mechanisms that prevent or assist in adapting to such taxing events. These defense mechanisms include the immune system, which is comprised of multiple molecular pathways that respond to traumatic stress. However, the timing and intensity of the immune reaction varies between individuals, especially during cardiothoracic surgeries. Thus, not every patient will have a smooth recovery post-op, or a ten-day hospital course when battling hospital-acquired pneumonia. This diversity of response will be highlighted in this discussion.

Each organ system of the human body has developed specific mechanisms for injury response. For example, stress to the skin may cause induration and erythema to an inflamed area, whereas the intestines often manifest inflammation by a change in bowel habits. This review will focus on the mechanisms of the heart and how the cardiac system responds to stress. Specifically, current data regarding biomarkers of inflammation will be examined to determine whether these markers can be used to predict clinical outcomes and overall patient survival.

The cardiovascular system is intricately extensive, and takes the brunt of many stress-inducing factors. According the American Heart Association and American Stroke Association in 2006, cardiovascular disease was the leading cause of mortality in the United States. While not every individual will endure a myocardial infarction, develop cardiomyopathy, or suffer from coronary artery disease, each person reacts to cardiovascular strain differently, and it is this aspect that provides the unique pathophysiology of the cardiovascular system.

One of the most common pathologies of the heart, coronary artery disease (CAD), is precipitated by the gradual deposition of fat and cholesterol to form atherosclerotic plaques in coronary vessels. The formation of these plaques reduces the diameter of the lumen of the vessels, thereby impairing antegrade myocardial blood flow. A stenotic coronary artery results in reduced coronary blood flow, and therefore decreased oxygen and nutrients delivered to the myocardium. When the heart is starved of oxygen and nutrients, it may become ischemic. This ischemia can be completely asymptomatic or manifest as angina. Prolonged ischemia may result in death of cardiac tissue, which is a permanent condition.

Depending on the severity of the coronary artery damage, patients may develop a partial blockage that may only result in transient ischemia and angina, or a complete blockage resulting in a myocardial infarction (MI). MI is defined as myocardial cell death due to prolonged ischemia [1]. MI is detected when blood levels of sensitive and specific biomarkers such as cTn (cardiac troponins I and T) or the MB fraction of creatine kinase (CKMB) are increased [1]. The trend of these biomarkers can indicate how acute the infarct is, the severity and if a re-infarct has occurred after the initial incident. This information is extremely helpful in clinical management of a patient with an infarct.

### Coronary artery bypass grafting

Numerous procedures are currently used to treat coronary blockages, including the use of tissue plasminogen activator (TPA), cardiac catheterization and coronary artery bypass grafting (CABG). During a CABG, a healthy artery or vein, typically the saphenous vein from the medial side of either lower extremity or the internal mammary artery from the chest wall, is grafted to bypass the blocked coronary artery. The three most commonly affected vessels are the left anterior descending artery (LAD), commonly known as the widow maker, the left circumflex artery, and the right coronary artery (RCA).

A CABG procedure usually takes 3 to 5 hours, and can include a single or multiple vessel bypass (Table 1). During the surgery, thoracic exploration is performed through a median sternotomy. Medication is administered to arrest the heart, an approach known as cardioplegia, which allows the surgeon to work on a stopped heart. The aorta is cross-clamped, and blood flow is then re-routed through the cardiopulmonary bypass machine during the procedure. After the case is completed and sinus rhythm of the heart is restored, the heart-lung bypass machine is removed.

**Table 1:** Different heart conditions and general characteristics (listed in decreasing order of occurrence)

**Condition**	**Length of operation**	**Mean age**	**Gender differences**	**Mortality rate**	**Treatment**	**Prevalence**
Coronary Artery Bypass Graft (CABG)	4-6 hours [2,3]	Usually >60 [3]	Majority occurs in males [4]	1.53 deaths per 1000 admissions [5]	Vein or artery used to create detour around blocked area in artery	About 427,000 operations performed each year [6]
						0.14%
Balloon angioplasty (Aortic coarctation)	30 minutes – 3 hours [7]	Congenital [2]	About 2:1 ratio, men to women [2]	1 in 1000 procedures [8]	Balloon placed inside aorta, then expanded [3]	Nearly one million performed per year in the US [9] (.003%)
Aortic Regurgitation	2-5 hours [2]	Prevalence increases with age, but severe AR is uncommon before 70 [10]	13% of men versus 8.5% of women [10]	<10% per year, once symptomatic [11]	Replacement of aortic valve [12]	Less than 1% of the general population [13]
				Death from any cause, once symptomatic: 4.7%		
				Death from aortic valve surgery: 14.6% [11]		
Aortic Stenosis	2-5 hours [2]	Can develop around 30 but mostly in patients 65 or older [14]	3 times more common in men than women [14]	Asymptomatic: <1% per year	Replacement of aortic valve [12]	Less than 200,000 people in US population [14]: .064%
				Symptomatic: 25% 1 year from onset of symptoms and 50% at 2 years[14]		
Atrial Septal Defect	2-3 hours [12]	Congenital [15]	3 times more common in women than in men [16]	Surgical repair in the first 2 decades of life is associated with a mortality rate near zero. [13]	Surgical closure of shunt	1.6 per 1000 live births [16] . 4,131,019 births in the US in 2009; [17,18]
			Female-to-male ratio: 2–1 [12]			About .05% of births in the US.
Tetralogy of Fallot	2-3 hours [19]	Congenital [20]	Unrelated [20]	<10% with surgery; without surgery, death usually occurs by age 20. [11]	Widen pulmonary tract and repair VSD	5 out of every 10,000 babies [21] = .05% of all babies
Hypoplastic left heart syndrome		Congenital; evidence of genetic inheritance [22]	67% males[22]	Lethal without surgery. Stage 1 survival: 68-77%. [23]	Parts of left side of heart do not develop fully	.16-.36 per 1000 live births [22]
(3 surgeries in total)						.026% of all live births
Heart Transplant	Anywhere from 4–12 hours, depending on the person [24]	48+/−11.8 years [25]	72.4% male [26-28]	Survival rate at 12 months: 88% for men, 77.2% for women [24]	Heart is replaced by donor heart	2163 performed in 2008 [24]
						0.00%
Aortic Graft (Aortic coarctation)		Congenital [29]. Usually performed on older patients, for whom surgery poses significant risk [26-28]	Greater in men than women [30]	success rate >95% [26-28]	Aorta replaced with synthetic aorta or patient’s artery	37, 102 procedures from 1986 to 2004 [31]
						About 1952 per year; 6.26e-4%
Anastomosis	Less than 30 minutes [31]	Congenital [2]		Mortality rate highest in neonates <1 week. Mortality rate during, or within one month, of surgery was 24% (50/208) and late mortality rate was 10% (21/208). [32]	Free ends of aorta are reconnected	83 patients between 1999 and 2000 [31]
						2.7e-5% of the general US population

An alternative procedure, off-pump CABG is performed on a beating heart. It is indicated for high-risk patients who are not stable enough to use the cardiopulmonary bypass machine for prolonged periods of time. These patients often have a history of peripheral vascular disease, cerebral vascular accidents (CVA), or multiple transient ischemic attacks (TIAs).

The CABG procedure is complex and does not come without risk. Cardiac surgery is a highly invasive procedure that can potentially lead to further myocardial insult. The degree of insult is dependent on multiple factors such as the type and extent of the surgical procedure, duration of cardiopulmonary bypass and aortic cross-clamping, pre-operative condition of the heart, length of anesthesia use and timing of post-operative revascularization [33].

Cardiac surgery with the cardiac bypass machine causes an acute inflammatory response through activation of inflammatory cells, complement cascade, and secretion of cytokines and chemokines. Vascular endothelial cells are activated and increase leukocyte adhesion molecules, a marker for inflammation. This same response also occurs once the cross-clamp is released and the heart is re-perfused [34]. It is understood that during the use of CPB, and after reperfusion, a rise in the production of free radicals occurs that causes damage to the vasculature, also known as reperfusion injury [35].

### Serum biomarkers

#### Protein biomarkers

A major biomarker in the heart is serum cardio-specific troponin T. Troponin T is one of three proteins of the troponin regulatory complex found on tropomyosin, a protein involved in the contractile apparatus of cardiac muscle. Troponin T is integral to muscle contraction in skeletal and cardiac muscle. Although typically present at low levels in the heart, its levels rise after cardiac surgery, before returning to baseline levels. Troponin T levels have been shown to correlate with the duration of cardioplegia [33]. Recently, a new highly sensitive and specific immunoassay to detect cardiac troponin T in the serum was introduced. A promising and potential application for this marker is better prediction of patient outcome and a clearer understanding of the extent of cardiac damage [36]. As stated above, inducing cardioplegia does not come without risk, and some patients are able to tolerate longer hours in cardioplegia than others before suffering repercussions. This assay will help identify the patients more susceptible to damage secondary to cardioplegia.

In addition to Troponin T and IL-6, cardiac troponin I (CTn1), myoglobin, creatine kinase (CK) and its isoenzyme creatine kinase-myocardial band (CK-MB), which is specific to the heart, are all involved in cardiac inflammation. Serum concentrations of these protein biochemical markers are increased during acute coronary syndrome (ACS), pericarditis, cardiac surgery and renal dysfunction [37-39]. CTn1, CK, CK-MB and myoglobin are released from damaged cardiac cells into the interstitial space. Serum concentrations of these biomarkers are elevated during cardiac surgery, renal dysfunction, and pericarditis [37]. Levels of cTn1 increased after use of the CPB machine [40].

In addition to C-reactive protein, tetranectin is a plasma protein found in secretory cells such as monocytes. Upon stimulation from stress or trauma, tetranectin facilitates monocyte migration to the site of injury. High concentrations of tetranectin are found in areas of regenerating muscle and are associated with tissue remodeling [41].

B-type natriuretic peptide (BNP), a protein commonly used to evaluate heart failure, is a polypeptide secreted by the ventricles of the heart in response to excessive stretching of cardiac myocytes. This molecule is responsible for decreasing systemic vascular resistance and central venous pressure as well as an increasing natriuresis. This adaptive response allows for the reduction of systemic blood pressure, decrease in afterload, and therefore increase in cardiac output. Measuring BNP is an indirect way to evaluate ventricular function and identify patients at risk that may be asymptomatic. Using this marker to stratify patients prior to CABG can help eliminate the need for additional testing. Although checking a patient’s BNP level is typically reserved for patients admitted for congestive heart failure exacerbation, with the current data indicating BNP as a marker for pre-op and post-op patient outcome, ordering a BNP for pre-op clearance may become more mainstream. Especially in patients with a history of CAD, increased BNP levels are associated with an increased rate of myocardial infarction and cardiovascular death during mid-term follow-up. BNP levels are a prognostic marker associated with higher mortality in patients with myocardial infarction, cardiogenic shock, and pulmonary embolism.

NT-proBNP, another established marker for cardiac failure, is also present in other pathologies, such as exacerbated chronic obstructive pulmonary disease, acute coronary syndromes, atrial fibrillation, and myocarditis. In contrast to BNP, higher NT-proBNP levels are associated with female gender, impaired renal function, and older age. NT-pro-BNP is one of the biomarkers increased by myocardial ischemia or subsequent, burdened wall stress.

#### Adhesion molecule biomarkers

Several adhesion molecules have also been identified to play essential roles as cardiac inflammation biomarkers.

E-selectin, also known as CD62 antigen-like family member E (CD62E), is an endothelial-leukocyte adhesion molecule. It plays an important part in recruiting leukocytes to a site of injury. The local release of cytokines by damaged cells induces the overexpression of E-selectin on endothelial cells of nearby blood vessels. As the inflammatory response progresses, chemokines released by injured tissue enter the blood and activate rolling leukocytes, which then tightly bind to the endothelial surface and exit the blood vessels via diapedesis. Leukocytes migrate through the interstitium to the site of injury or infection guided by chemotactic signals. The levels of E-selectin were found to be the highest in the right atrium and the lowest in the left ventricle [42].

#### Cytokine biomarkers

Certain cytokines have been discovered to be closely related to the inflammatory cascade and also can be used as biomarkers to trend response to cardiac stress. While patients are on the CPB machine, pro-inflammatory cytokines such as tumor necrosis factor alpha (TNF-α) and interleukins (IL) 1, 6 and 8 are released and mediate the systemic inflammatory response syndrome (SIRS), which is believed to play an essential role in myocardial ischemia and reperfusion injury [43]. Table 2 describes some of these and other markers. Comparing the extent of elevation of these markers among patients will help ascertain those that are more at risk for reperfusion injury. IL-6 is a polypeptide secreted by activated monocytes and endothelial cells that initiates the acute phase of the inflammatory cascade and decreases cardiac contractility. This decline in cardiac output secondary to decrease in contractility is a response seen in patients who are in shock. This also leads to a drop in blood pressure. It is this decrease in perfusion that leads to damage of the myocardium. The level of IL-6 steadily increases during CABG, reaching its peak in the 6^th^ hour of the procedure. This indicates that patients with a higher amount of IL-6 at hour 6 might suffer from greater drops in cardiac contractility, thus a decrease in cardiac output and lower blood pressure and therefore insufficient circulation to the myocardium.

**Table 2:** Various inflammatory biomarkers (Gene) and brief descriptions

**Gene biomarker**	**Description**	**Increased/Decreased/Remained constant after surgery**
S-TnT	Part of the troponin complex	Increased to 1.26 μg/L1 day after surgery; decreased to .52 μg/L 4 days after surgery [33]
NSE	Enzyme; levels increase after brain damage	Rises to 20.2 ng/mL postoperatively but returns to normal levels 48 h postop [44]
HSP 70	Helps with protein folding and transport	Increased to about 2000 pg/mL during surgery but decreased to 0 by 19 hrs postop [45]
TLRs	Signaling receptors for HSPs	Decreased during operation but increased postop [45]
TNF-α	Quantity correlated to the quantity of HSP70	Increased in both MUF and non-MUF groups during surgery; normalized 24 h postop [40]
IL-1ra	Inflammatory cytokine	Clear rise in both MUF and non-MUF groups. Reached peak 6 hrs postop in both groups but returned to baseline levels 12 hrs postop in MUF group, while staying at a higher level in the non-MUF group [40]
IL-8	Neutrophil chemotactic and activating factor	Levels did not change much in group with SIRS but increased to about 46 pg/mL in the group with MODS and continued to decrease after [46]
IL-18	Induce chemokines. Involved in inflammatory response following extracorporeal circulation	Remained constant in group with SIRS but increased to 300 pg/mL 1 day postop and remained about that level in group with MODS [46]
IL-1β	Biologically inactive precursor molecule	In the MUF group, IL-1β rose to max levels post-op. Fell significantly post-op and remained at these levels. IL-1β levels remained constant [40]
IL-6	Quantity correlated to the quantity of HSP70	Levels rose after surgery to about 100 pg/mL and remained through days 1 and 2 after surgery [47]

#### Coagulation cascade biomarkers

While the coagulation cascade may not appear to be directly related to inflammation, the risk of coagulopathic events increases when inflammation is present, and this attribute can be used as a marker for stress. Anti-thrombin 3 is a substance in blood plasma that inactivates thrombin during the clotting cascade, and is elemental in preventing post-operative deep vein thrombosis (DVT) and pulmonary embolism (PE). Its levels fell significantly post-op [40], indicating that risk for developing DVT or PE status post-CABG is increased. This is strong evidence promoting the importance of anticoagulation status post-CABG and early ambulation.

The Von Willebrand factor (vWF) protein is involved in the intrinsic pathway of the coagulation cascade. It is bound to factor VIII and activated by thrombin. This helps to induce the binding of platelets and eventually clot formation. Increased plasma levels of vWF in a large number of cardiovascular, neoplastic and connective tissue diseases are presumed to arise from adverse changes to the endothelium, and may contribute to an increased risk of thrombosis.

#### Complement cascade biomarkers

### Breath biomarkers

While a variety of hematological markers are used to monitor pre-op and post-op status, less invasive techniques have also been utilized. An excellent study performed by Pabst et al. determined that oxidative and metabolic stress during cardiac surgery could be monitored by breath biomarkers. They assessed acetone, isoprene, and pentane, and found that acetone release was an indicator of stress and isoprene levels correlated with cardiac output. Acetone in the breath mirrors other metabolic stresses, such as diabetic ketoacidosis [23]. The increased acetone is secondary to stress-induced release of catecholamines, which cause increased lipolysis and release of cortisol. This cortisol surge results in increased dextrose turnover. Concentrations of ketone bodies are increased when dextrose metabolism is impaired and lipolysis is triggered, and eventually acetone is generated. There is a positive relationship between exhaled acetone levels and post-operative troponin T, as well as pre-op CRP and exhaled acetone levels. The correlation between breath acetone level and clinical condition underlies the potential of using breath biomarker monitoring for diagnostic methods and timely initiation of life-saving therapy [23].

Pabst et al. also assessed reactive oxygen species (ROS) and the release of ethane and pentane during peroxidation of omega 3 and omega 6 fatty acids. During CABG, exhaled pentane levels decreased immediately after the sternotomy was performed. Exhaled pentane concentrations measured immediately after termination of extracorporeal circulation correlated with the CK:CKMB ratio measured a few hours later, suggesting that exhaled pentane might serve as a fast and direct biomarker for early recognition of oxidative stress and ischemic reperfusion injury. Together this suggests that pentane acts as a fast and direct biomarker [23].

### Biomarkers in outcomes studies

Numerous studies have investigated pre-operative and post-operative variables that influence the outcome of CABG patients. Evaluating factors such as co-morbidities, family history, and diet can help anticipate problems that may ensue during the procedure or obstacles that might impede recovery time. While these aspects are very influential with regard to determining patient outcome, asking patients about family history and diet does not always guarantee an accurate answer. The use of biomarkers to predict prognosis serves as a more concrete method and eliminates variables that could alter the data.

Fernandez et al. demonstrated that patients with recent or ongoing ischemia exhibited higher preoperative cardiac troponin I, CK, CKMB, and myoglobin concentrations in pericardial fluid than in serum [37]. Possible mechanisms involved in this pericardial accumulation of specific and non-specific myocardial markers include increased leaks of cytoplasmic proteins due to dysfunction of the cardiomyocyte membrane and augmented vascular and epicardial permeability. As a consequence of myocardial ischemia, the integrity of the cell membrane is compromised, and cytosolic proteins may be released into the myocardial interstitium, accounting for protein leakage from the heart into the pericardial cavity. In addition, heightened capillary and pericardial permeability may increase the macromolecular exchange of tissue and plasma proteins [37].

Eikvar et al. compared post-CABG levels of cardiac markers in groups of patients that suffered from post-operative MI and those that did not [33]. The first post-operative day patients with postoperative myocardial infarction (POMI) had higher levels of serum troponin T and CK-MB compared to those that did not suffer from a POMI. At day 4, the difference in serum troponin T was more pronounced. The finding made by Katus et al. that the duration of cardioplegia correlates with the levels of serum troponin T has provided much needed information regarding the use of troponin T as an essential tool for evaluating the degree of myocardial damage [48]. Measuring these levels peri-operatively may aid in anticipating the risk of POMI and perhaps in its prevention. If elevated levels of serum troponin T and CK-MB prior to CABG are found, risk stratification can aid in pre-op planning and optimizing for surgery. Enforcing the importance of weight loss, exercise, medication compliance and exercise may result in a more positive post-op course.

BNP is secreted primarily by the muscle cells of the pumping chambers in the heart as a response to increased stress. Elevated levels of postoperative BNP during the first several days after CABG surgery is known to be associated with increased mortality. However, further research has analyzed whether BNP is also associated with poorer long-term physical function.

A study performed by Morimoto et al. found that plasma levels of BNP are markedly elevated in the acute phase after cardiac surgery requiring bypass and reflect the left ventricular function at the same time. Furthermore, myocardial damage due to ischemia may participate in the mechanism of synthesis and secretion of BNP [49]. The study examined the concentration of BNP immediately after CABG and followed the course during cardiac rehabilitation (CR). After CABG, the level of BNP was elevated, with the highest value immediately post-op, and showed a downwards trend following the procedure.

A study performed on CABG surgery patients at Brigham and Women’s Hospital in Boston and the Texas Heart Institute in Houston both pre- and post-operatively focused on levels of BNP and post-op outcome. Increased levels of postoperative BNP were found to be significantly associated with poor physical function six months to two years status post-CABG.

Wazni et al. found that patients with atrial fibrillation following cardiac surgery exhibited higher BNP levels than patients who remained in sinus rhythm throughout the post-operative course [50]. When considering that CABG patients with higher BNP levels are at an increased risk for both mortality and poor post-op outcome, it is imperative that these patients are considered for early and aggressive medical surveillance and treatment programs aimed at reducing BNP levels [51].

Both BNP and its counterpart, the N terminal fragment of proBNP, are established markers for cardiac failure. Schachner et al. determined the influence of preoperative serum NT-proBNP on postoperative outcome and mid-term survival in patients undergoing coronary artery bypass grafting (CABG) [52]. In general, patients with elevated pre-op NT-ProBNP exhibited a higher rate of complications. Patients with NT-proBNP levels greater than 502 ng/l had more co-morbidities than those with NT-proBNP levels less than 502 ng/l. Postoperatively, those patients had a significantly longer time before they were able to be weaned from the vent, a longer ICU stay, a higher rate of renal failure requiring hemofiltration, a higher rate of IABPs, and a higher rate of postoperative atrial fibrillation. Atrial fibrillation is a common complication after cardiac surgery. Although it is easily manageable with rate controlling agents and anticoagulation, if uncontrolled it can affect cardiac output and put patients at risk for CVA.

Preoperative measurement of NT-proBNP levels can be used to determine CABG patients with an increased risk as shown above. Patients with increased NT-proBNP should have close follow-up and periodic monitoring. Mayer et al. found that CABG patients with NT-proBNP levels > 862 pmol exhibited decreased long-term survival rates without clinical manifestation of heart failure. Therefore, elevated preoperative serum NT-proBNP levels are associated with a higher postoperative early and mid to long-term mortality, as well as morbidity, in patients undergoing isolated CABG [52].

vWF is a protein found in the endothelium that plays an essential role in platelet adhesion, and functions as a biomarker for endothelial dysfunction and damage. vWF was shown to be a predictor of ischemic heart disease in healthy individuals and patients with angina or acute MI [53]. Certain intra-operative variables, such as the extent of the surgical procedure, duration of use of the bypass machine, and amount of ischemia from cross clamping, are reported to be higher in patients who developed post-operative MI [33]. In patients with elevated vWF, special consideration can be taken in length of cross-clamping and duration on CPB machine. This finding of elevated vWF was also associated with blood type O. As all patients are typed and screened prior to CABG to prepare blood for transfusion, patients with blood type O could also be screened for vWF for risk assessment and modification. If those with an initially high vWF level are at risk for post-op MI and ischemic stroke [54], certain precautions should be taken in light of the increased chance of poor outcome.

CPB has generally been associated with increased levels of plasma soluble adhesion molecules of the endothelium, which have been attributed to the activation and injury of endothelial cells. Thus, the availability of useful biochemical markers for evaluating the endothelial state post-operatively offers a useful, non-invasive means to assess return of the endothelium to the unperturbed state, which is important in improving patient outcome [53]. Among the relevant markers, E and P selectins were found to be the most influential. These adhesion molecules mediate the initial steps of weak and reversible leukocyte diapedesis. They play a role in the migration of activated leukocytes into the interstitium where they degranulate and promote inflammation. Elevated levels of E and P selectin have been found in patients suffering from hyperlipidemia, PAD (peripheral artery disease), and CAD. High levels of these selectins reflect platelet activation and induce procoagulation activity associated with vascular thrombotic disease. Plasma selectins have been shown to predict major cardiovascular events in patients with pre-existing PAD or CAD [53].

While it is useful to study a certain patient population before, during, or after a CABG procedure, it can also be very helpful to focus on the same population throughout the entire process. Panagiotopoulous et al. measured concentrations of E and P selectins, tetranectin, von Willebrand Factor, and angiotensin converting enzyme in patients undergoing CABG prior to and up to 3 days after their procedure [53]. These markers were compared with patient demographics and multiple perioperative characteristics [53]. Differences in levels among markers between patients correlated with the degree of pre-existing endothelial damage and atherosclerosis.

Panagiotopoulos et al. assessed persistence of elevated biomarker levels in the post-operative CABG period. The post-operative period begins in the first moments when the patient’s chest is closed, and the patient is extubated and taken to the cardiac surgical intensive care unit. This is a critical period, during which the patient’s vitals and overall clinical performance must be closely monitored to avoid complications. Panagiotopoulos et al. concluded that of all molecules exhibiting elevated levels, tetranectin (TN) remained elevated on the 3^rd^ day post-operatively in the majority of patients, while other markers decreased [53]. This could be explained by the fact that tetranectin is found in areas of tissue remodeling and muscle repair.

### Preventing inflammation

The purpose of using biomarkers to predict post-operative outcomes of cardiac patients is to improve survival and quality of life. Other methods have been developed that help prevent adverse outcomes, such as the use of heparin. Heparin is a highly sulfated glycosaminoglycan produced by endothelial cells that functions as an innate anticoagulant. It can be injected as an anticoagulant, administered intravenously, and used to form an inner anticoagulant surface on various experimental and medical devices, such as test tubes and dialysis machines.

A study performed by Ljunghusen et al. assessed the level of inflammation between two groups of CABG patients, one for which the bypass machine was heparinized and one for which it was not [55]. C3a, as well as MAC-1 (macrophage-1 antigen), was increased in the group that was treated using the non-heparinized machine. The results demonstrated that heparin coating increased biocompatibility, resulting in less activation of complement and a reduction in circulating monocytes and granulocytes during the procedure [55]. There was a reduction in the number of granulocytes after five minutes of bypass, and a highly significant reduction in granulocytosis evident the morning after the procedure, suggesting that an increase in expression of L-selectin was found in the group with heparinized bypass but not in the group without heparin.

As mentioned above, the use of heparinized systems not only aids in a reduction of granulocytosis but also affects the expression of HLA-DR. The percentage of monocytes expressing HLA-DR inversely correlated with the risk of developing postoperative infections following major resectional surgery as well as survival after severe trauma. There was no difference in the expression of HLA-DR during or after bypass surgery, but HLA-DR expression was lower the morning after surgery in patients with heparinized than in non-heparinized CPB systems (Table 3). L-selectin levels were elevated in both groups during bypass. Granulocyte cell counts decreased during bypass, but normalized after the procedure. C3a desArg is a degradation product of C3a, both of which reflect complement activation during inflammatory response and disease. C3a mediates inflammatory reactions such as smooth muscle contraction, histamine release from mast cells, vasodilatation, and chemotaxis of eosinophils.

**Table 3:** Various inflammatory biomarkers (Serum) and brief descriptions

**Serum biomarker**	**Description**	**Increased/Decreased/Remained constant after surgery**
S-CKMB	Rise in amount in blood indicates that muscle damage has occurred	Increased to 40 μg/L1 day after surgery; decreased to 3 μg/L 4 days after surgery [33]
CD11b/CD18	Adhesion protein	No significant difference between SNP/non-SNP patients[32]; Expression of CD11b increased in group with heparin coating [37]
HLA-DR	Major histocompatibility complex	Significantly lower morning after operation [37]
CD62L	L-selectin	Increased during surgery but normalized the morning after [37]
Monocytes	Activation triggers production of cytokines	Decreased to 448 × 10^6^ cells/L during surgery; increased to 1889 × 10^6^ cells/L the next morning [37]
C3a desArg	Mediates inflammatory reactions	Increased in noncoated group during surgery; levels decreased the morning after surgery [37]
Granulocytes	A type of white blood cells categorized by the presence of granules	Reduction of circulating granulocytes noted in both groups after surgery; increased the morning after [37]
ATIII	Inactivates thrombin	Levels fell significantly in groups with and without MUF filtration. Reached baseline values 12 h postop.[40]
F1 + 2	Prothrombin fragment	Rose significantly in both MUF and non-MUF groups. Fell to baseline values 6 h postop. [40]
CXC /CC-chemokine receptors	Act mainly on neutrophils and lymphocytes	Postoperative expression of CXCR2 but not CXCR1 was significantly lower in patients with cyanotic vs. noncyanotic heart lesions [56,57]
GCP-1	Granulocyte chemotactic protein	Levels remained unchanged during study period [56]
ENA-78	Epithelial neutrophil-activating peptide	Decreased from 908 pg/mL to 729 pg/mL during surgery and remained at low levels 3 h after surgery (376 pg/mL) [56]
MPO	Marker of primary neutrophil granules	Increased from 11.3 ng/mL before surgery to 45.4 ng/mL during surgery and remained at that level 3 hrs after surgery [56]
PCT	Hormone produced by the thyroid	Increased to 17 ng/mL 1 day postop and decreased throughout the next 3 days [46]
Pre-albumin	Anti-acute phase protein synthesized in liver	Significantly lower during operation in cold priming group; levels were almost normalized 24 hrs after surgery [35]
HSCRP	Highly sensitive C-reactive protein	Levels rose 30 fold by 24 hrs post-op in cold priming group and 18 fold by 24 hrs post-op in the warm priming group [35]
α-1 antitrypsin	Protease inhibitor: inhibits enzymes that bind peptides together	Decreased by half during surgery then increased 24 hrs postop in cold priming group; decreased by half during surgery in the warm priming group also, but increased 3 fold 24 hrs postop [35]

The use of modified ultrafiltration (MUF) has proven to be helpful in achieving electrolyte balance in fluid overloaded patients as well as in ridding inflammatory mediators associated with the use of the cardiopulmonary. MUF has been shown to decrease transfusion requirements and improve post-operative oxygenation and myocardial contractility in children after CPB [40]. However, whether the reduction in inflammatory response is due to decreases in the pro-inflammatory cytokines, increases in anti-inflammatory cytokines, or both remains unknown.

In addition to MUF, sodium nitroprusside (SNP) has also been found to help in regaining homeostatis after using the CPB machine. SNP was discovered to reduce reperfusion injury in experimental studies. It has multiple physiological effects, such as blocking free radicals, inhibiting platelet aggregation, regulating membrane permeability, and decreasing the effects of leukocytes. During the reperfusion period of a CABG, the production of nitric oxide (NO) is decreased, and this induces the expression of adhesion molecules, such as the membrane attack complex. Elevated levels of the membrane attack complex result in increased neutrophil adhesion to the endothelium. SNP was found to be effective at controlling hypertension during CPB, and its use decreases the need for vasodilators in the early post-operative period [34]. SNP has a cardio-protective effect attributed to its induction of nitric oxide synthase resulting in production of NO. NO reduces reperfusion injury in experimental studies, and has been shown to block super oxide radicals, inhibit platelet aggregation and adhesion, regulate the permeability of the endothelial layer, and inhibit the function of leukocytes.

A study was performed with two groups of patients undergoing CPB: the control group received a 5% dextrose solution after the release of the aortic cross-clamp, and the SNP group was given 0.5 μg/kg/min SNP [34]. No significant difference was found between groups in the expression of CD11b/CD18 (Table 3). Significant increases of IL-6 were detected three hours after the termination of CPB, with a significant difference between groups at that point. There were significant increases in both groups in neutrophil counts, and a gradual decrease of platelet counts in both groups during the procedure. During CPB, vasodilator infusion for the control of hypertension was not needed for any of the patients in the SNP group, but was needed for 50% of patients in the control group. During their ICU stay, patients received vasodilators for 39.2 ± 20.6 hours in the control group vs. 6.4 ± 10.3 hours in the SNP group. Patients in the SNP group stayed significantly shorter in the ICU: 28.2 ± 9.3 vs. 53.0 ± 15.9 hours. No blood analysis abnormalities were detected in any of the groups in the early post-operative period, but one patient in the control group expired secondary to ventricular arrhythmia resulting in decreased cardiac output on post-operative day 3 [34].

Adenosine was found to have a cardio-protective effect during open heart surgery. Adenosine could be used in ischemic preconditioning (IPC) as an adjunct to cross-clamping the aorta. Compared with simple cold blood cardioplegia in heart valve replacement patients, adenosine pretreatment (100 μg/kg/min, 10 min) as an adjunct to 1 mM adenosine cold blood cardioplegia may reduce CTnI, IL-6, and IL-8 release, resulting in reduced myocardial injury after surgery [43]. The leakage of these cytokines causes post-op myocardial dysfunction and damage. Adenosine A1 receptors and possibly A3 receptors are also known to confer protection through inhibitory G protein coupled pathways and receptor activation on cardioprotective agents such as mitochondria [43].

Buyukates et al. found that the use of warm priming solution in open heart surgery resulted in significant improvements in acute inflammatory markers [35]. Levels of highly sensitive C-reactive protein (HSCRP) increased 10–2,000 fold within 6–12 hours, and α-1 antitrypsin increased 2–5 fold within 24 hours (Table 3). The use of warm priming solution also had favorable outcomes in hemodynamic parameters of the patients during and after the surgery, such as lowest mean arterial blood pressure (MABP) during cross clamping and highest MABP after cross clamping, and more frequent spontaneous beating of the heart following removal of the cross clamp. The 36°C warm priming solution prevented sudden and rapid changes in the systemic circulation, resulting in marked improvement in the systemic inflammatory response and more stable hemodynamic status [35].

When considering the markers discussed above, attempts made to lower these values peri-operatively by exercise, change in diet, medication or other mechanisms may prove to be beneficial in improving patients’ post-op course. We discovered that using heparinized machines, modified ultrafiltration and warm priming solution in anticipation of possible adverse events has aided in more positive post-op courses. If modifying pre-op factors would help reduce unfavorable outcomes, then further evaluating the markers of inflammation may prove to be beneficial. In conjunction with these methods to prevent inflammation or adverse effects, measurements of BNP, troponin and CRP levels, for example, several months prior to CABG would provide helpful information and the possibility of decreasing these markers and altering post-CABG outcomes.

## Conclusions

Previous studies have reported biomarkers that were elevated or depressed during the CABG procedure or post-operatively. However, the majority of these studies did not investigate correlations between levels of markers and patient outcome. While we know that a rise in certain markers indicates that the cardiac system has been stressed, it is not clear if high levels indicate and correlate with more stress, and therefore a worse recovery course. Our objective was to examine the literature on cardiac biomarkers and identify associations regarding outcome from the current published data. Expression of the monocyte marker, HLA DR, was found to be inversely proportionate to the risk for developing post-operative infections. HLA DR may prove to be useful if examined pre-operatively, and could possibly be used as an indicator of risk of post-operative infection. An important correlation was made between exhaled pentane concentrations measured after coming off the CPB machine. This non-invasive biomarker may aid with early recognition of oxidative stress and ischemic reperfusion injury. Early awareness and treatment of tissue damage can help prevent further injury. High levels of P and E selectins may also predict major cardiovascular events in patients with pre-existing PAD or CAD [23]. This can be helpful when considering pre-operative work up labs, specifically for patients with a past medical history of heart disease. Von Willebrand Factor may be useful as a biomarker for endothelial dysfunction and damage. The prospect of developing the means to predict ischemic heart disease in healthy individuals and patients with angina or acute MI is exciting, and could potentially alter medical care in the future [23]. The use of biomarkers is fairly well established in other fields of medicine, such as oncology, however is not well explored in predicting cardiac patient prognosis. Our hope is that biomarkers will become routinely used in this field and will help to improve patient outcome.

## Abbreviations

ACS: Acute coronary syndrome; ATIII: Anti-thrombin 3; C3a: Mcomplement component 3; CABG: Coronary artery bypass graft; CAD: Coronary artery disease; CD11b/CD18: Cluster of differentiation 11b/ cluster of differentiation 18; CD62E: E-selectin; CD62L: I-selectin; CK: Creatine kinase; CK-MB: Creatine kinase-myocardial band; CPB: Cardiopulmonary bypass; CPK: Creatine phosphokinase; CTn1: Cardiac troponin 1; DVT: Deep vein thrombosis; ENOS: Endothelial nitric oxide synthase; F1 + 2: Prothrombin fragment; HLA-DR: Human leukocyte antigen-d related; HSCRP: Highly-sensitive C-reactive protein; ICAM-1: Intercellular adhesion molecule-1; Il-6: Interleukin-6; IPC: Ischemia preconditioning; LAD: Left anterior descending (artery); MABP: Mean arterial blood pressure; MAC-1: Macrophage-1 antigen; MI: Myocardial infarction; NO: Nitrous oxide; PAD: Peripheral artery disease; PE: Pulmonary embolism; POMI: Postoperative myocardial infarction; RC: Right coronary (artery); ROS: Reactive oxygen species; SIRS: Systemic inflammatory response syndrome; SNP: Sodium nitroprusside; TIA: Transient ischemic attack; TN: Tetranectin; vWF: von Willebrand factor.

## Competing interests

The authors declare that they have no competing interests.

## Authors’ contributions

IP, as the primary author, helped draft the main manuscript. RG performed writing, editing, organization, formatting, and literature review. KJ performed much of the research and literature search. AA, LL and EE reviewed the manuscript for errors and provided input related to cardiothoracic surgery. As the corresponding author, KS designed the review, supervised the data gathering and literature search, and participated in the editing of the manuscript. All authors read and approved the final manuscript.
